# Computed tomography findings and prognosis in older COVID-19 patients

**DOI:** 10.1186/s12877-022-02837-7

**Published:** 2022-03-01

**Authors:** Chukwuma Okoye, Panaiotis Finamore, Giuseppe Bellelli, Alessandra Coin, Susanna Del Signore, Stefano Fumagalli, Pietro Gareri, Alba Malara, Enrico Mossello, Caterina Trevisan, Stefano Volpato, Gianluca Zia, Fabio Monzani, Raffaele Antonelli Incalzi

**Affiliations:** 1 Geriatrics Unit, Department of Clinical and Experimental Medicine, University of Pisa, Pisa, Italy; 2 Geriatrics Unit, Department of Medicine, Campus Bio-Medico University and Teaching Hospital, Rome, Italy; 3 School of Medicine and Surgery, Acute Geriatric Unit, University of Milano-Bicocca, San Gerardo Hospital, Monza, Italy; 4 Geriatrics Unit and the GeroCovid Working Group, Department of Medicine (DIMED), University of Padua, Padua, Italy; 5 Bluecompanion Ltd, London, UK England; 6 Geriatric Intensive Care Unit, Department of Experimental and Clinical Medicine, University of Florence, Florence, Italy; 7 Center for Cognitive Disorders and Dementia - Catanzaro Lido, ASP Catanzaro, Italy; 8 ANASTE Humanitas Foundation, Rome, Italy; 9 Department of Medical Sciences, University of Ferrara, Ferrara, Italy

**Keywords:** SARS-CoV-2, Oldest, Old, Tomography, X-ray computed, Pleural

## Abstract

**Background:**

In older and multimorbid patients, chronic conditions may affect the prognostic validity of computed tomography (CT) findings in COVID-19. This study aims at assessing to which extent CT findings have prognostic implications in COVID-19 older patients.

**Methods:**

Hospitalized COVID-19 patients aged 60 years or more enrolled in the multicenter, observational and longitudinal GeroCovid study who underwent chest CT were included. Patients were stratified by tertiles of age and pneumonia severity to compare CT findings. Hierarchical clustering based on CT findings was performed to identify CT-related classificatory constructs, if any. The hazard ratio (HR) of mortality was calculated for individual CT findings and for clusters, after adjusting for potential confounders.

**Results:**

380 hospitalized COVID-19 patients, with a mean age of 78 (SD:9) years, underwent chest CT scan. Ground glass opacity (GGO), consolidation, and pleural effusion were the three most common CT findings, with GGO prevalence decreasing from younger to older patients and pleural effusion increasing. More severe the pneumonia more prevalent were GGO, consolidation and pleural effusion. HR of mortality was 1.94 (95%CI 1.24–3.06) for pleural effusion and 13 (95%CI 6.41–27) for cluster with a low prevalence of GGO and a high prevalence of pleural effusion (“LH”), respectively. Out of the three CT based clusters, “LH” was the only independent predictor in the multivariable model.

**Conclusions:**

Pleural effusion qualifies as a distinctive prognostic marker in older COVID-19 patients. Research is needed to verify whether pleural effusion reflects COVID-19 severity or a coexisting chronic condition making the patient at special risk of death.

**Trial registration:**

ClinicalTrials.gov: NCT04379440

**Supplementary Information:**

The online version contains supplementary material available at 10.1186/s12877-022-02837-7.

## Background

COVID-19 qualifies as a systemic disease with a great variety of symptoms and damaged organs. However, it basically remains a respiratory disease in the vast majority of patients. Indeed, respiratory symptoms and gas exchange indexes have primary classificatory and prognostic implications. CT scan findings have also been repeatedly reported to contribute to the diagnosis and prognostic classification of COVID-19 [1]. In a noteworthy proportion of cases, CT findings could supplement microbiologic exams and correctly address the diagnosis [2, 3]. CT abnormalities have been frequently reported in asymptomatic patients [4], but completely normal CT findings have been observed in mild cases [5, 6]. Indeed, the earlier CT signs do not always correlate with symptom severity, and CT abnormalities, even severe, have been frequently reported to be relatively common in asymptomatic patients. However, the CT pattern of interstitial pneumonia and its typical expressions, like ground glass opacities (GGO), interlobular septal thickening, and reticular pattern, which were initially associated with COVID-19, is only one of the many CT findings. Atypical CT findings, like pleural effusions, nodules, multifocal pneumonia have been well described [7, 8].

This bulk of evidence clearly states that CT scan plays a primary role in diagnosing and monitoring COVID-19. However, the possible confounding effect of conditions such as chronic obstructive pulmonary disease (COPD) or congestive heart failure (CHF) in the interpretation of CT findings has not been tested so far in older and multimorbid patients. Furthermore, combined and not isolated CT findings might play a major classificatory and prognostic role. Thus, assessing clustered rather than individual CT findings might improve our understanding of CT findings’ clinical meaning. These two topics are the object of the present study. Based on the GeroCovid data, we will provide an estimation of the real-life CT findings’ contribution to the prognostic stratification in older and multimorbid COVID-19 inpatients.

## Methods

### Study Population

GeroCovid Observational is a multi-purpose and multicenter initiative promoted by the Italian Society of Gerontology and Geriatrics (SIGG) in collaboration with Bluecompanion that aims at investigating the impact of SARS-CoV-2 pandemics in older patients in different settings of care. The objectives of the project are setting specific. In the GeroCovid acute wards cohort, we purposed to assess COVID-19 clinical presentation and course and identify the prognostic factors of the disease in older people. Data are collected retrospectively and prospectively in an e-Registry since March 1^st^, 2020. The enrolment of new cases ended on December 31^st^, 2020, and the follow-up will be closed on June 30^th^, 2021. Overall, 66 investigational sites are actively participating. The final endpoint of GeroCovid is to provide high-quality and comprehensive data, which will help optimize COVID-19 prevention and management of patients > 60 years. GeroCovid conforms to the principles outlined in the Declaration of Helsinki, it was registered at ClinicalTrials.gov (NCT04379440) and the participation of each centre was authorized by the corresponding local Ethical Committee [9] (see e-Appendix for the full list). All methods were performed in accordance with the relevant guidelines and regulations.

### Data collection

Inpatients aged 60 or older who underwent chest CT examination within 48 h from hospitalization and positive reverse transcriptase- polymerase chain reaction test (RT-PCR) for SARS-CoV-2 from March 1^st^, 2020 to December 31^st^, 2020 were retrospectively studied, without any exclusion criteria. All the patients received routine CT scan according to the local institutional protocol, and the CT findings retrieved from the radiological reports were utilized for the final analysis. The number of pulmonary lobes involved was used as a severity score. Demographics, comorbidity, clinical and laboratory data were collected for all patients, including a comprehensive geriatric assessment (CGA). The presence of frailty was also evaluated according to modified Fried criteria [10]. Participants’ clinical status at the start and end of hospitalization were divided into five-clinical category ordinal scale referred to hospitalized patients according to the classification recommended by the WHO R&D Blueprint expert group [11], which has already been used in several studies in COVID-19 patients [12–14], and: 1) patients not requiring oxygen therapy; 2) patients requiring oxygen by mask or nasal prongs. 3) with high-flow oxygen or non-invasive ventilation (HF/NIV); 4) needing intubation and mechanical ventilation; 5) death. The severity of respiratory failure was assessed by calculating the PaO_2_/FIO_2_ ratio [15] (i.e., partial pressure arterial oxygen/fraction of inspired oxygen ratio) of the first arterial blood gas analysis performed at ward admission (in the case of using Venturi mask, the FIO_2_ indicated in the swivel connector was utilized) [15]. Primary endpoints were: “death”, “clinical improvement”, defined as the improvement of at least 2/5 point from admission to discharge or as being discharged alive from the hospital, whichever came first, and “no major change at discharge”, defined as no change from admission to discharge at the same on the five-category ordinal scale. Additionally, the transfer to a different hospital was recorded.

### Statistical analysis

For the purposes of this analysis, patients who did not undergo chest CT or did not in time were excluded (see e-Figure. 1). Descriptive statistics were used to report participants’ characteristics: mean and standard deviation (SD) or median and interquartile range (IQR), after checking for normality distribution, for continuous variables, and frequency and percentage for categorical variables. The study population was categorized by tertiles of age: Group 1 [60–74 years], Group 2 [75–83 years], and Group 3 [≥ 84 years]. The clinical, laboratory, and radiological findings of these age groups were compared using ANOVA or Kruskal–Wallis test for continuous variables, as appropriate, and chi-squared test for categorical variables.

**Fig. 1:**
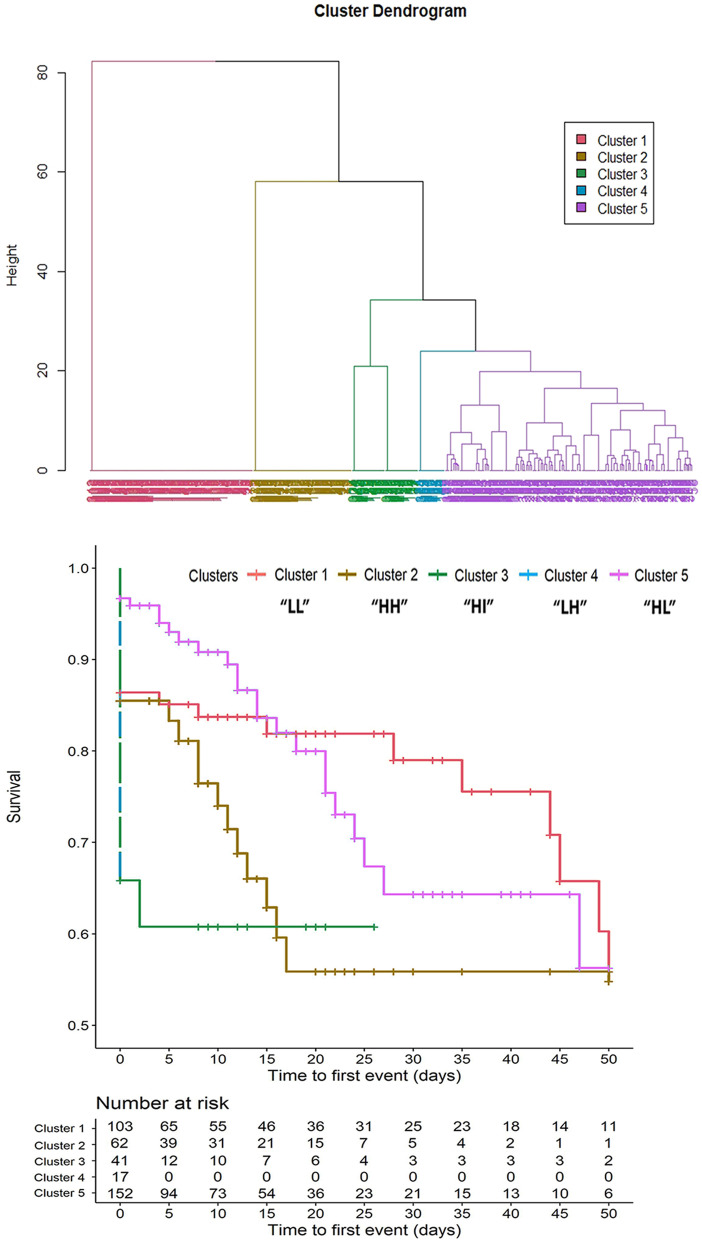
Clusters of Computed Tomography findings (above) and their survival curves during hospitalization (below)

In order to detect potential classificatory and prognostic constructs inaccessible to an age-based classification of the patients, hierarchical clustering was performed on CT scan data using an agglomerative nesting algorithm (i.e. AGNES). Ward’s method was used as a linkage criterion. The result of clustering was represented using a dendrogram. Five clusters were identified according to the dendrogram and their radiological, clinical, and laboratory characteristics were compared as previously described. The risk of mortality was evaluated using a Kaplan Meier estimator. After checking the proportional hazards assumption using Schöenfeld residuals, the hazard ratio (HR) and 95% confidence interval (95%CI) of mortality was calculated for each CT scan finding and cluster, using the cluster with the lowest risk as a reference. Univariable and multivariable models were performed, the latter being adjusted for age, sex, BMI, frailty, and those diseases with a well-known impact on the CT scan and outcome, i.e. CHF, COPD, and diabetes. Results are represented using a forest plot. All statistics were performed using R version 4.0.2 (The R Foundation for Statistical Computing, Vienna, Austria, 2020) using the packages “cluster”, “dendextend”, “survival” and “surviminer”.

## Results

Three hundred eighty hospitalized patients with COVID-19 underwent chest CT. The mean age was 78 (SD:9) years, 56% were males, and three quarters were living at home before hospital admission. At baseline, according to pneumonia severity 47% was treated with Oxygen by mask or nasal prongs, 14% with HF/NIV and 5% was intubated and mechanically ventilated. GGO prevalence at CT scan was 85%, consolidation 41%, pleural effusion 23%, pulmonary nodules 12%, and bronchiectasis 9%; all other CT findings had a prevalence of 5% or lower. Images of the three most common CT-findings are reported in e-Figure. 2. The most common outcomes were clinical improvement (52%) and death (25%) (see Table 1). Excluded inpatients were older (mean age:79 vs 78 years), had lower PaO_2_/FiO_2_ ratio (mean:250 vs 271), higher prevalence of cognitive impairment (22% vs 13%), shorter hospital stay (median days:3 vs 8) and greater mortality (31% vs 25%) (see e-Table 1).

**Fig. 2:**
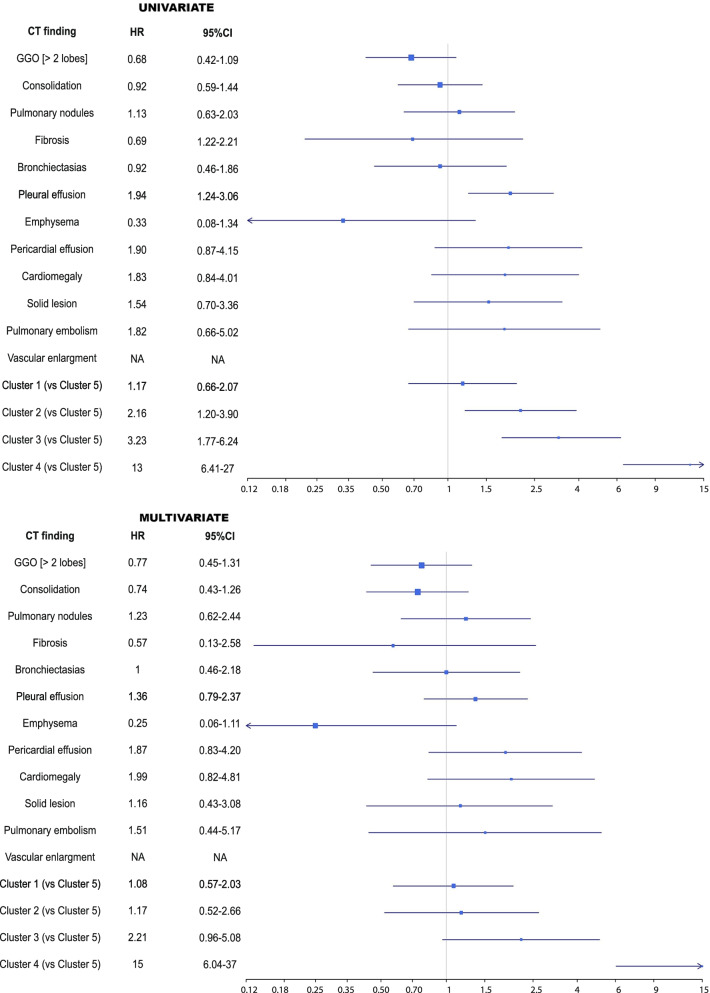
Hazard ratios for the associations between radiologic findings and in-hospital mortality at the univariate (above) and multivariate (below) Cox regression models

**Table 1:** Characteristics of the population stratified by tertiles of age

	**Population (*n *:380)**	**Group 1** **Aged 60–74 (*n *:139)**	**Group 2** **Aged 75–83 (*n *:116)**	**Group3** **Aged ≥ 84 (*n *:125)**	*P*-value
**Age (years)**	78(9)	68.(4)	79(2)	89(4)	-
**Gender (M)**	214(56)	99(71)	62(53)	53(42)	< 0.001
**Household situation**					
Living at home	281(74)	115(83)	94(81)	72(58)	< 0.001
**Nutritional status**					
Obese	60(16)	31(22)	18(16)	11(9)	0.01
Underweight/malnourished	48(13)	5(4)	14(12)	19(15)	< 0.001
**PaO**_**2**_**/FIO**_**2**_	271 (110)	265 (112)	282 (116)	267 (102)	0.12
**Frailty**	31(8)	9(6)	9(8)	13(10)	0.49
**Pneumonia severity**					
No oxygen therapy	128(34)	54(39)	38(33)	36(29)	0.22
Oxygen by mask or nasal prongs	179(47)	49(36)	60(52)	70(57)	0.002
NIV or HF oxygen	52(14)	22(16)	13(11)	17(14)	0.56
Intubation and mechanical ventilation	17(5)	12(9)	5(4)	0(0)	0.003
**Main comorbidities**					
Diabetes mellitus	83(22)	27(19)	32(28)	24(19)	0.20
Chronic heart failure	40(11)	9(6)	12(10)	19(15)	0.07
Atrial fibrillation	50(13)	6(4)	19(16)	25(20)	< 0.001
Cognitive impairment	51(13)	4(3)	17(15)	30(24)	< 0.001
Hypertension	83(22)	33(24)	28(24)	22(18)	0.37
Chronic kidney disease	50(13)	11(8)	18(16)	21(17)	0.07
COPD	38(10)	7(5)	15(13)	16(13)	0.05
**Hospital stay (days)**	8 (IQR:19)	6.5 (IQR:17.2)	8.5 (IQR:19.8)	8 (IQR:21)	0.67
**CT scan**					
GGO	322(85)	128(92)	97(84)	97(78)	0.004
Consolidation	156(41)	56(40)	52(45)	48(38)	0.57
Pulmonary nodules	47(12)	9(6)	24(21)	14(11)	0.001
Fibrosis	18(5)	5(4)	6(5)	7(6)	0.68
Bronchiectasis	34(9)	12(9)	11(9)	11(9)	0.93
Pleural effusion	86(23)	23(17)	21(18)	42(34)	0.001
Emphysema	19(5)	4(3)	5(4)	10(8)	0.12
Cardiomegaly	19(5)	2(1)	5(4)	12(10)	0.01
Pericardial effusion	19(5)	3(2)	5(4)	11(9)	0.03
Solid lesion	19(5)	3(2)	10(9)	6(5)	0.05
Pulmonary embolism	10(3)	3(2)	4(3)	3(2)	0.75
Subsegmental vascular enlargement	1(0)	0(0)	0(0)	1(0)	0.35
**Outcome**					
Clinical improvement	198 (52)	88 (63)	66 (57)	44 (35)	< 0.001
Death	95 (25)	19 (14)	24 (21)	52 (42)	< 0.001
No major change	14 (4)	6 (4)	4 (3)	4 (3)	0.88
Transfer to a different hospital	71 (18)	24 (17)	22 (19)	25 (20)	0.85

Data are reported as mean *SD* or median *IQR* and frequency %, as appropriated. Abbreviations: *PaO2*/*FIO2* Partial pressure arterial oxygen/fraction of inspired oxygen ratio, *NIV* Non-invasive ventilation, *HF H*igh-flow, *COPD *Chronic obstructive pulmonary disease, *GGO *Ground glass opacity

Group 1 (60–74 years), Group 2 (75–83 years) and Group 3 (≥ 84 years) were composed by 139, 116 and 125 patients, respectively. The prevalence of men significantly decreased from Group 1 to Groups 3, as well as the percentage of individuals living at home, while the percentage of underweight/malnourished individuals increased. Mortality was significantly higher in Group 3 and, although no difference was found among groups in PaO_2_/FIO_2_, no patient in Group 3 was intubated and mechanically ventilated. The prevalence of GGO progressively decreased from Group 1 to Group 3, while that of pleural effusion, cardiomegaly, and pericardial effusion increased. Of note, there was no difference in the prevalence of pleural effusion (21% versus 28%, *P*-value 0.53), cardiomegaly (12% versus 6%, *P*-value 0.35) and pericardial effusion (6% versus 6%, *P*-value 0.99) between participants with and without chronic heart failure. Furthermore, no difference was found for consolidation and other considered findings. Participants with consolidation did not differed from those without in terms of C-reactive protein – 245 mg/l versus 271 mg/l, *P*-value 0.58 – and procalcitonin – 10 ng/ml versus 11 ng/ml, *P*-value 0.47 –, laboratory tests suggestive of sepsis. More details about the study population are reported in Table 1. GGO were more frequently localized in the lower lobes (see e-Table 2). In the whole sample, GGO and consolidation prevalence progressively increased with the severity of COVID-19 pneumonia, with all patients intubated and mechanically ventilated having GGO and 65% having consolidation. Stratifying by age groups, the association between CT scan findings and pneumonia severity waned (see Table 2).

**Table 2:** Distribution of Computed Tomography scan findings according to hospital admission COVID-19 pneumonia severity stratification

CT scan	COVID-19 pneumonia disease severity stratification				*P*-value
	**No oxygen therapy** **(*n *:128)**	**Oxygen by mask or** **nasal prongs** **(*n *:179)**	**Non-invasive ventilation or** **high-flow oxygen** **(*n *:52)**	**Intubation and mechanical** **ventilation** **(*n *:17)**	
**GGO**	104(81)	152(85)	47(90)	17(100)	0.05
Aged 60–74 (*n*:139)	47(87)	46(94)	22(100)	12(100)	0.04
Aged 75–83 (*n*:116)	28(74)	52(87)	12(92)	5(100)	0.18
Aged ≥ 84 (*n*:125)	29(81)	54(77)	13(76)	0(0)	0.78
Frailty (*n*:31)	8(57)	12(86)	2(100)	1(100)	0.25
**Consolidation**	43(36)	71(40)	28(54)	11(65)	0.07
Aged 60–74 (*n*:139)	17(31)	17(35)	13(59)	7(58)	0.05
Aged 75–83 (*n*:116)	12(32)	28(47)	8(62)	4(80)	0.27
Aged ≥ 84 (*n*:125)	14(39)	26(37)	7(42)	0(0)	0.97
Frailty (*n*:31)	7(50)	7(58)	2(100)	1(100)	0.47
**Pulmonary nodules**	16(13)	23(13)	6(12)	0(0)	0.20
Aged 60–74 (*n*:139)	2(4)	3(6)	3(14)	0(0)	0.10
Aged 75–83 (*n*:116)	9(24)	12(20)	3(23)	0(0)	0.65
Aged ≥ 84 (*n*:125)	5(14)	8(11)	0(0)	0(0)	0.18
Frailty (*n*:31)	4(29)	4(36)	0(0)	0(0)	0.67
**Fibrosis**	3(2)	12(7)	3(6)	0(0)	0.35
Aged 60–74 (*n*:139)	2(4)	3(6)	1(5)	0(0)	0.94
Aged 75–83 (*n*:116)	1(3)	4(2)	1(8)	0(0)	0.78
Aged ≥ 84 (*n*:125)	0(0)	6(9)	1(6)	0(0)	0.34
Frailty (n:31)	0(0)	2(20)	1(50)	0(0)	0.12
**Bronchiectasis**	16(13)	10(6)	5(10)	2(12)	0.27
Aged 60–74 (n:139)	5(9)	3(6)	1(5)	2(17)	0.32
Aged 75–83 (n:116)	5(13)	4(7)	2(9)	0(0)	0.42
Aged ≥ 84 (n:125)	6(17)	3(4)	2(12)	0(0)	0.17
Frailty (*n*:31)	2(14)	2(22)	0(0)	0(0)	0.83
**Pleural effusion**	19(15)	47(26)	13(25)	4(24)	0.04
Aged 60–74 (*n*:139)	3(6)	11(22)	5(23)	2(17)	< 0.01
Aged 75–83 (*n*:116)	5(13)	12(20)	2(15)	2(40)	0.49
Aged ≥ 84 (*n*:125)	11(31)	24(34)	6(35)	0(0)	0.98
Frailty (*n*:31)	0(0)	1(10)	1(50)	0(0)	0.67
**Emphysema**	6(5)	7(4)	5(10)	1(6)	0.56
Aged 60–74 (*n*:139)	2(4)	1(2)	0(0)	1(8)	0.73
Aged 75–83 (*n*:116)	0(0)	2(3)	3(23)	0(0)	< 0.01
Aged ≥ 84 (*n*:125)	4(11)	4(6)	2(12)	0(0)	0.69
Frailty (*n*:31)	0(0)	2(22)	0(0)	0(0)	0.25
**Pericardial effusion**	1(1)	17(9)	1(2)	0(0)	0.01
Aged 60–74 (*n*:139)	0(0)	3(6)	0(0)	0(0)	0.16
Aged 75–83 (*n*:116)	0(0)	5(3)	0(0)	0(0)	0.23
Aged ≥ 84 (*n*:125)	1(3)	9(13)	1(6)	0(0)	0.30
Frailty (*n*:31)	0(0)	1(10)	0(0)	0(0)	0.58
**Cardiomegaly**	3(2)	8(4)	7(13)	1(6)	0.01
Aged 60–74 (*n*:139)	0(0)	0(0)	1(5)	1(8)	0.16
Aged 75–83 (*n*:116)	1(3)	3(5)	1(8)	0(0)	0.80
Aged ≥ 84 (*n*:125)	2(6)	5(7)	5(29)	0(0)	0.04
Frailty (*n*:31)	0(0)	1(11)	0(0)	0(0)	0.62
**Solid lesion**	7(5)	7(4)	5(10)	0(0)	0.43
Aged 60–74 (*n*:139)	0(0)	1(2)	2(9)	0(0)	0.18
Aged 75–83 (*n*:116)	6(16)	2(3)	2(15)	0(0)	0.07
Aged ≥ 84 (*n*:125)	1(3)	4(6)	5(29)	0(0)	0.43
Frailty (*n*:31)	0(0)	1(11)	0(0)	0(0)	0.62
**Pulmonary embolism**	3(2)	4(2)	2(4)	1(6)	0.86
Aged 60–74 (*n*:139)	0(0)	1(2)	1(5)	1(8)	0.47
Aged 75–83 (*n*:116)	2(5)	2(3)	0(0)	0(0)	0.80
Aged ≥ 84 (*n*:125)	1(3)	1(1)	1(6)	0(0)	0.78
Frailty (*n*:31)	0(0)	0(0)	0(0)	0(0)	-
**Subsegmental vascular enlargement**	0(0)	1(1)	0(0)	0(0)	0.88
Aged 60–74 (*n*:139)	0(0)	0(0)	0(0)	0(0)	-
Aged 75–83 (*n*:116)	0(0)	0(0)	0(0)	0(0)	-
Aged ≥ 84 (*n*:125)	0(0)	1(1)	0(0)	0(0)	0.84
Frailty (*n*:31)	0(0)	0(0)	0(0)	0(0)	-

Data are reported as frequency (%). Abbreviations: *GGO* Ground glass opacity

CT scan-based clustering identified five clusters (see Fig. 1) with the following radiological characteristics according to the three most common CT scan findings.

Cluster 1 showed a low prevalence of both GGO and pleural effusion (“LL”), Cluster 2 a high prevalence of both the previous findings (“HH”), Cluster 3 a high prevalence of GGO and intermediate prevalence of pleural effusion (“HI”), Cluster 4 a low prevalence of GGO and high of pleural effusion (“LH”) and Cluster 5 a high prevalence of GGO and low of pleural effusion (“HL”). Clusters were similar in terms of consolidation, except for Cluster 4 (“LH”) showing a low prevalence. More details on CT-scan findings by clusters are reported in e-Table 3.

**Table 3:** Distribution of clinical characteristics by clusters based on Computed Tomography -scan findings

	**Cluster 1 (*n *:103)** **“LL”**	**Cluster 2 (*n *:62)** **“HH”**	**Cluster 3 (*n *:42)** **“HI”**	**Cluster 4 (*n *:17)** **“LH”**	**Cluster 5 (*n *:156)** **“HL”**	*P*-value
**Age (years)**	81 (8)	81 (11)	76 (10)	80 (8)	76 (9)	< 0.001
**Gender (M)**	46(45)	27(44)	27(64)	6(35)	108(69)	< 0.001
**Household situation**						
Living at home	87(84)	31(50)	23(55)	8(47)	132(85)	< 0.01
**Nutritional status**						
Obese	18(20)	8(13)	9(21)	3(18)	22(14)	0.66
Underweight/malnourished	18(20)	6(10)	7(16)	3(18)	14(9)	0.26
**PaO**_**2**_**/FIO**_**2**_	311(108)	260(103)	261(120)	202(108)	257(106)	0.001
**Pneumonia severity**						
No oxygen therapy	33(32)	21(34)	17(41)	5(29)	52(34)	0.89
Oxygen by mask or nasal prongs	63(62)	31(50)	16(38)	10(59)	59(39)	< 0.01
NIV or HF oxygen	6(6)	8(13)	6(14)	2(12)	30(19)	0.05
Intubation and mechanical ventilation	0(0)	2(3)	3(7)	0(0)	12(8)	0.04
**Main comorbidities**						
Diabetes mellitus	20(19)	15(24)	7(17)	3(18)	38(24)	0.74
Chronic heart failure	16(16)	6(10)	2(5)	2(12)	14(9)	0.32
Atrial fibrillation	14(14)	6(10)	8(19)	1(6)	21(13)	0.60
Cognitive impairment	16(16)	15(24)	5(12)	2(12)	13(8)	0.04
Hypertension	20(19)	2(3)	8(19)	3(18)	50(32)	0.001
Chronic kidney disease	13(13)	11(18)	6(14)	4(24)	16(10)	0.41
COPD	9(9)	4(6)	2(5)	2(12)	21(13)	0.35
**Hospital stay (days)**	11 (IQR:28.5)	9.5 (IQR:16.8)	0 (IQR:9)	0 (IQR:0)	9 (IQR:16)	< 0.01
**Outcome**						
Clinical improvement	58(56)	27(44)	21(50)	4(24)	88(56)	0.04
Death	22(21)	19(31)	17(40)	12(71)	25(16)	< 0.01
No major change	2(2)	0(0)	1(2)	0(0)	11(7)	0.06
Transfer to a different hospital	20(19)	15(24)	3(7)	1(6)	32(21)	0.13

Data are reported as mean *SD* or median *IQR* and frequency (%), as appropriated. Abbreviations: *PaO2/FIO2* Partial pressure arterial oxygen/fraction of inspired oxygen ratio, *NIV* Non-invasive ventilation, *HF* High-flow, *COPD* Chronic obstructive pulmonary disease

Furthermore, clusters differed in PaO_2_/FIO_2_ and the outcome. Cluster 1 (“LL”) had a PaO_2_/FIO_2_ > 300, suggesting a less severe respiratory impairment, which is confirmed by the infrequent use of HF/NIV, Cluster 2 (“HH”), 3 (“HI”) and 5 (“HL”), sharing a high prevalence of GGO, had a PaO_2_/FiO_2_ of around 250, with more severe pneumonia frequently treated with HF/NIV or intubation, and Cluster 4 (“LH”) had the lowest PaO_2_/FIO_2_, at the threshold for acute respiratory distress syndrome, with the most severe disease. The last cluster showed significantly higher mortality, with Cluster 1 (“LL”) and Cluster 5 (“HL”) having the lowest values. A comparison of clinical and laboratory characteristics between different clusters can be found in Table 3.

Pleural effusion was the only single CT finding associated with the outcome (HR 1.94, 95%CI 1.24–3.06). Likewise, Cluster 2 (“HH”), Cluster 3 (“HI”), and Cluster 4 (“LH”) showed an increased HR of death. After adjusting for confounders, neither CT scan findings alone nor clusters, except Cluster 4 (“LH”), were significantly associated with the outcome (see Fig. 2).

## Discussion

The present study confirms that GGO is the most common CT pulmonary alteration in older COVID-19 inpatients [16, 17]. Instead, the prevalence of pleural effusions, bronchiectasis, and pulmonary nodules was higher than in the studies carried out in younger cohorts of hospitalized patients with COVID-19. Noteworthy, the presence of pleural effusion at admission qualified as a marker of disease severity.

It has been extensively reported that chest CT imaging can show typical radiological findings of COVID-19 even before the onset of clinical symptoms [2, 3], helping clinicians identify COVID-19 patients who initially had negative RT-PCR results [18]. Older patients may present atypical signs [19] of SARS-CoV-2 infection, with clinical presentations like fever and cough less frequently prevailing [20], while delirium may be the only clinical sign of COVID-19 in the absence of any other symptoms [21, 22]. Therefore, CT is extremely helpful for the diagnosis and follow-up of COVID-19 in older patients due to its high sensitivity [2]. Our analysis confirmed GGO as the most common CT pulmonary alteration in our cohort of older inpatients, more typically localized in the lower left lobe, in line with several previous studies [16]. Similarly, we detected a high proportion of bilateral GGO involvement, and one-fourth of the cohort presented a diffuse GGO pattern with lesions represented in all lung lobes. Our results are consistent with previous studies, which reported GGO with bilateral pulmonary involvement as the predominant CT manifestation, even in asymptomatic cases of COVID-19 patients [4]. Interestingly, we found a higher proportion of pleural effusions, bronchiectasis, and pulmonary nodules—which are not typically associated with COVID-19 pneumonia—compared to the previous evidence [16]. In 2020, Wang et al. [23] found pleural effusion in 4.9% of cases, while two recent meta-analysis [16, 17] yielded a prevalence of 5.6% and 5.8%, respectively. In the current study, we detected a higher proportion of pleural effusion with an upward age-related trend: while in the youngest age-group, one-fifth of the patients showed pleural effusion, the prevalence doubles in the oldest old, yielding a 35%. Interestingly, chronic conditions like congestive heart failure and renal failure, both potential causes of pleural effusion [24], were more common in the oldest age tertile. Thus, in these patients pleural effusion might be a marker of vulnerability related to the underlying chronic condition or, else, an effect of COVID-19. Noteworthy, in non COVID-19 populations pleural effusion was associated with a 50%, 25% and 46% increased 1-year mortality rate in case of heart failure, hepatic failure and renal failure, respectively [25]. Similarly, we detected an overall 9% prevalence of bronchiectasis, higher than in previous studies [26], that might reflect the age-related increase in the frequency of bronchiectasis in the general population and might have clinical implications since bronchiectasis has been associated with Aspergillosis overinfection [27].

To our knowledge, no previous studies on COVID-19 pneumonia have dealt with differences in CT scan findings across age groups in older patients. Data from the US Centers for Disease Control and Prevention [28] reveal that the risk of dying from COVID-19 increases approximately tenfold for every 20 years of age; thus, thorough investigations on the oldest old are needed. Zhu et al. [29] compared CT findings of patients younger and older than 60 years old, but the mean age of the older cohort (68.4 ± 6.0) was lower than in the current study. Comparably, the mean age of inpatients in the study of Wang et al. was 57.6 ± 15.7. Both the studies concluded that older patients having more lobes affected and subpleural lesions were more likely to present a higher disease severity compared to younger counterparts.

It is to some extent surprising that GGO were less frequently found in the oldest old patients, although individuals aged 84 or older showed a higher mortality compared to younger counterparts. This rather contradictory result may be due to several possible reasons. First, in selected older patients COVID-19 might be only an incidental finding in the context of a severe or life-threatening condition like end -stage CHF. This might also contribute to explain the prognostic role of pleural effusion. Alternatively, a too timely CT scan might have missed late onset CGO; the well-known delayed immunologic response to acute stimuli in the very old supports this hypothesis [30]. Another possible explanation might be that even a mild systemic injury could be detrimental to the oldest old patients by decompensating a labile homeostasis, underlying the crucial role of frailty in COVID-19 mortality and morbidity, as recently demonstrated [31,32]. Nevertheless, this finding has implications in the clinical practice: in the oldest old, the absence of GGO should not rule out the diagnosis of COVID-19; rather, in case of suspicion, RT-PCR and repeat CT scan after a few days remain strictly recommended. Furthermore, in the light of this result, various validated GGO-based scores of pneumonia severity [33, 34] might be inaccurate in describing COVID-19 pneumonia in the oldest individuals.

Interestingly, the presence of pulmonary consolidation at admission did not emerge as an independent predictor of death; indeed, so far, contradictory results have been observed, with some studies reporting an association between consolidation and mortality, while others failed to show this relationship [35].

The hierarchical cluster analysis identified five different clusters according to CT scan findings at admission; in particular, two clusters (“LL” and “HL”) revealed a better outcome compared to the others. These two clusters presented the lowest prevalence of pleural effusion, yet additional factors might have contributed to a higher survival rate. For example, the majority of patients in “LL” and “HL” clusters were living at home, indirectly suggesting lower disability degree which may have impacted survival. Furthermore, Kaplan Meier curves highlight that while “HL” has a constant mortality rate over the first month, “LL” mortality is steeper in the first week and then declines. Interestingly, even though “HI” and “HL” clusters showed the same median age, they shaped two different phenotypes of COVID-19 patients; older persons in the “HL” cluster were less frequently obese (14% vs 21%) compared to the “HI” cluster. These characteristics might have played a role in mortality specifically in the first period of hospitalization, when most “HI” patients died, compared to “HL” cluster that showed a longer length of stay and a lower mortality.

Confirming the important meaning of pleural effusion, the “LH” cluster showed the lowest PaO_2_/FIO_2_ ratio and the higher mortality risk, resulting independently associated with death even after adjustment for comorbidities. Noteworthy, “LH” patients, alike patients in the “HI” cluster, showed a steep mortality curve, that is a very early mortality.

This study has several strengths. First, it is one of the largest studies of hospitalized geriatric patients with COVID-19 focusing on CT scan findings, with more than 100 patients aged more than 83 years old. Second, data are representative of real-life. Finally, to the best of our knowledge, this is the first study evaluating the prognostic impact of CT scan findings, alone or grouped in clusters, in old hospitalized patients with COVID-19 adjusting by age, frailty and chronic comorbidities. However, there are some limitations. First, due to variability in practice, many patients did not undergo CT scan. As a fact, missing data might have broadened the generalizability of the current investigation. Nonetheless, both cohorts shared in the majority the same “mild ARDS” category as defined in the Berlin Definition [36] and no differences were found between the two populations in terms of main comorbidities. Secondly, we evaluated the findings of the baseline CT scan, thus we did not assess the pattern of disease progression. Thirdly, pulmonary embolism was searched for through the iodine contrast only in cases judged worth of assessment, thus its prevalence is likely underestimated. Fourthly, stemming from a non-supervised hierarchical analysis, results of cluster analysis must be interpreted with caution, since specific findings could be influenced by the small sample size of the subgroups. Finally, in line with the GeroCovid protocol, all the CT scan reports were consecutively collected in e-Registry without a revision by a second blinded radiologist. However, this issue was linked to the real-life nature and real practice representativeness of our data.

## Conclusions

This study advances our knowledge about the prevalence and prognostic implications of CT findings in older COVID-19 patients. It discloses a changing prevalence of CT findings with age and allows reconsider the prognostic role of these findings at variance with adult populations, emphasizing the negative prognostic implications of pleural effusion.

## Supplementary Information

**Additional file 1:** Summary of the IRB approvals.

**Additional file 2:** **e-Figure 1**. CONSORT flowchart diagram. **e-Figure 2**. Common CT-scan findings in older patients with COVID-19 pneumonia. **e-Table 1.** Characteristics of the GeroCovid population included (n:380) and excluded (n:820) from the analysis. **e-Table2**. Ground glass opacities distribution stratified by tertiles of age. **e-Table 3**. Distribution of CT-scan findings by clustersm.

## Data Availability

The datasets used and/or analysed during the current study are available from the corresponding author on reasonable request.

## Abbreviations

CT: Computed tomography
GGO: Ground glass opacities
COPD: Chronic Obstructive Pulmonary Disease
CHF: Chronic Heart Failure
LL: Low prevalence of GGO, Low prevalence of pleural effusion
HH: High prevalence of GGO, High prevalence of pleural effusion
LH: Low prevalence of GGO, High prevalence of pleural effusion
HL: High prevalence of GGO, Low prevalence of pleural effusion
HI: High prevalence of GGO, Intermediate prevalence of pleural effusion

## References

[1] Caruso D, Zerunian M, Polici M, Pucciarelli F, Polidori T, Rucci C (2020). Chest CT Features of COVID-19 in Rome, Italy. Radiology.

[2] Ai T, Yang Z, Hou H, Zhan C, Chen C, Lv W (2020). Correlation of Chest CT and RT-PCR Testing for Coronavirus Disease 2019 (COVID-19) in China: A Report of 1014 Cases. Radiology.

[3] Long C, Xu H, Shen Q, Zhang X, Fan B, Wang C (2020). Diagnosis of the Coronavirus disease (COVID-19): rRT-PCR or CT?. Eur J Radiol..

[4] Shi H, Han X, Jiang N, Cao Y, Alwalid O, Gu J (2020). Radiological findings from 81 patients with COVID-19 pneumonia in Wuhan, China: a descriptive study. Lancet Infect Dis.

[5] Xu YH, Dong JH, An WM, Lv XY, Yin XP, Zhang JZ (2020). Clinical and computed tomographic imaging features of novel coronavirus pneumonia caused by SARS-CoV-2. J Infect.

[6] Zhang R, Ouyang H, Fu L, Wang S, Han J, Huang K (2020). CT features of SARS-CoV-2 pneumonia according to clinical presentation: a retrospective analysis of 120 consecutive patients from Wuhan city. Eur Radiol.

[7] Bao C, Liu X, Zhang H, Li Y, Liu J (2020). Coronavirus Disease 2019 (COVID-19) CT Findings: A Systematic Review and Meta-analysis. J Am Coll Radiol JACR.

[8] Ye Z, Zhang Y, Wang Y, Huang Z, Song B (2020). Chest CT manifestations of new coronavirus disease 2019 (COVID-19): a pictorial review. Eur Radiol.

[9] Trevisan C, Del Signore S, Fumagalli S, Gareri P, Malara A, Mossello E, et al. Assessing the impact of COVID-19 on the health of geriatric patients: The European GeroCovid Observational Study. Eur J Intern Med. 2021;87:29–35. 10.1016/j.ejim.2021.01.017 PMC7847394 33573885

[10] Pedone C, Costanzo L, Cesari M, Bandinelli S, Ferrucci L, Antonelli IR (2016). Are Performance Measures Necessary to Predict Loss of Independence in Elderly People?. J Gerontol A Biol Sci Med Sci.

[11] R&D Blueprint and COVID-19 [Internet]. Available from: https://www.who.int/teams/blueprint/covid-19. Accessed 9 Apr 2021.

[12] Beigel JH, Tomashek KM, Dodd LE, Mehta AK, Zingman BS, Kalil AC (2020). Remdesivir for the Treatment of Covid-19 — Final Report. N Engl J Med.

[13] Goldman JD, Lye DCB, Hui DS, Marks KM, Bruno R, Montejano R (2020). Remdesivir for 5 or 10 Days in Patients with Severe Covid-19. N Engl J Med.

[14] Cao B, Wang Y, Wen D, Liu W, Wang J, Fan G (2020). A Trial of Lopinavir-Ritonavir in Adults Hospitalized with Severe Covid-19. N Engl J Med.

[15] Boatright JJM, Hess DMIN (2020). Therapeutic gases: management and administration. Respiratory Care: Principles and Practice.

[16] Muhammad SZ, Ahmed A, Shahid I, Khalid A, Menezes RG, Sheikh MU (2020). Chest computed tomography findings in hospitalized COVID-19 patients: a systematic review and meta-analysis. Infez Med.

[17] Garg M, Gupta P, Maralakunte M, Kumar M P, Sinha A, Kang M (2021). Diagnostic accuracy of CT and radiographic findings for novel coronavirus 2019 pneumonia: Systematic review and meta-analysis. Clin Imaging.

[18] Young BE, Ong SWX, Kalimuddin S, Low JG, Tan SY, Loh J (2020). Epidemiologic Features and Clinical Course of Patients Infected With SARS-CoV-2 in Singapore. JAMA.

[19] Kimball A, Hatfield KM, Arons M, James A, Taylor J, Spicer K (2020). Asymptomatic and Presymptomatic SARS-CoV-2 Infections in Residents of a Long-Term Care Skilled Nursing Facility — King County, Washington, March 2020. MMWR Morb Mortal Wkly Rep.

[20] Perry A, Macias Tejada J, Melady D (2018). An Approach to the Older Patient in the Emergency Department. Clin Geriatr Med.

[21] Kennedy M, Helfand BKI, Gou RY, Gartaganis SL, Webb M, Moccia JM (2020). Delirium in Older Patients With COVID-19 Presenting to the Emergency Department. JAMA Netw Open.

[22] Rebora P, Rozzini R, Bianchetti A, Blangiardo P, Marchegiani A, Piazzoli A (2021). Delirium in Patients with SARS-CoV-2 Infection: A Multicenter Study. J Am Geriatr Soc.

[23] Wang J, Zhu X, Xu Z, Yang G, Mao G, Jia Y (2020). Clinical and CT findings of COVID-19: differences among three age groups. BMC Infect Dis.

[24] Debiasi EM, Pisani MA, Murphy TE, Araujo K, Kookoolis A, Argento AC (2015). Mortality among patients with pleural effusion undergoing thoracentesis ERJ Express. Eur Respir J.

[25] Walker SP, Morley AJ, Stadon L, De Fonseka D, Arnold DT, Medford ARL (2017). Nonmalignant Pleural Effusions: A Prospective Study of 356 Consecutive Unselected Patients. Chest.

[26] Salehi S, Abedi A, Balakrishnan S, Gholamrezanezhad A (2020). Coronavirus Disease 2019 (COVID-19): A Systematic Review of Imaging Findings in 919 Patients. AJR Am J Roentgenol.

[27] Benedetti MF, Alava KH, Sagardia J, Cadena RC, Laplume D, Capece P (2021). COVID-19 associated pulmonary aspergillosis in ICU patients: Report of five cases from Argentina. Med Mycol Case Rep.

[28] Demographic Trends of COVID-19 cases and deaths in the US reported to CDC [Internet]. Available from: https://covid.cdc.gov/covid-data-tracker/#demographics. Accessed Apr 2021.

[29] Zhu T, Wang Y, Zhou S, Zhang N, Xia L (2020). A Comparative Study of Chest Computed Tomography Features in Young and Older Adults With Corona Virus Disease (COVID-19). J Thorac Imaging.

[30] Cunha LL, Perazzio SF, Azzi J, Cravedi P, Riella LV (2020). Remodeling of the Immune Response With Aging: Immunosenescence and Its Potential Impact on COVID-19 Immune Response. Frontiers in Immunology.

[31] Sinclair AJ, Abdelhafiz AH. Age, frailty and diabetes – triple jeopardy for vulnerability to COVID-19 infection. EClin Med. 2020;22:100343. 10.1016/j.eclinm.2020.100343 PMC7177130 32328575

[32] Zhou F, Yu T, Du R, Fan G, Liu Y, Liu Z (2020). Clinical course and risk factors for mortality of adult inpatients with COVID-19 in Wuhan, China: a retrospective cohort study. Lancet.

[33] Saeed GA, Gaba W, Shah A, Al Helali AA, Raidullah E, Al Ali AB (2021). Correlation between Chest CT Severity Scores and the Clinical Parameters of Adult Patients with COVID-19 Pneumonia. Radiol Res Pract.

[34] Yang R, Li X, Liu H, Zhen Y, Zhang X, Xiong Q (2020). Chest CT Severity Score: An Imaging Tool for Assessing Severe COVID-19. Radiol Cardiothorac Imaging.

[35] Hashemi-madani N, Emami Z, Janani L, Khamseh ME (2021). Typical chest CT features can determine the severity of COVID-19: A systematic review and meta-analysis of the observational studies. Clin Imaging.

[36] Ranieri VM, Rubenfeld GD, Thompson BT, Ferguson ND, Caldwell E, Fan E (2012). Acute respiratory distress syndrome: the Berlin Definition. JAMA.

