# Supplementary material for: Computed tomography findings and prognosis in older COVID-19 patients
Source: BMC Geriatr. 2022 Mar 1;22:166. doi: 10.1186/s12877-022-02837-7 (PMC8885320; doi:10.1186/s12877-022-02837-7)

**Computed tomography findings and prognosis in COVID-19 older patients**

Chukwuma Okoye^1^, Panaiotis Finamore^2^, Giuseppe Bellelli^3^, Alessandra Coin^4^, Susanna Del Signore^5^, Stefano Fumagalli^6^, Pietro Gareri^7^, Alba Malara^8^, Enrico Mossello^6^, Caterina Trevisan^4^, Stefano Volpato^9^, Gianluca Zia^5^, Fabio Monzani^1^, Raffaele Antonelli Incalzi^2^

^1^ Geriatrics Unit, Department of Clinical and Experimental Medicine, University of Pisa, Pisa, Italy

^2^ Geriatrics Unit, Department of Medicine, Campus Bio-Medico University and Teaching Hospital, Rome, Italy

^3^ School of Medicine and Surgery, Acute Geriatric Unit, University of Milano-Bicocca, San Gerardo Hospital, Monza, Italy

^4^ Geriatrics Unit and the GeroCovid Working Group, Department of Medicine (DIMED), University of Padua, Italy

^5^ Bluecompanion Ltd, London, UK

^6^ Geriatric Intensive Care Unit, Department of Experimental and Clinical Medicine, University of Florence, Italy

^7^ Center for Cognitive Disorders and Dementia - Catanzaro Lido, ASP Catanzaro, Italy

^8^ ANASTE Humanitas Foundation, Rome, Italy

^9^ Department of Medical Sciences, University of Ferrara, Ferrara, Italy

**Corresponding Author:**

Panaiotis Finamore, MD PhD

Unit of Geriatrics,

Campus Bio Medico University and Teaching Hospital

Via Alvaro del Portillo, 200 – Rome Italy

Ph. +39 06-225411167

e-mail: p.finamore@unicampus.it

**Summary of the IRB approvals.**


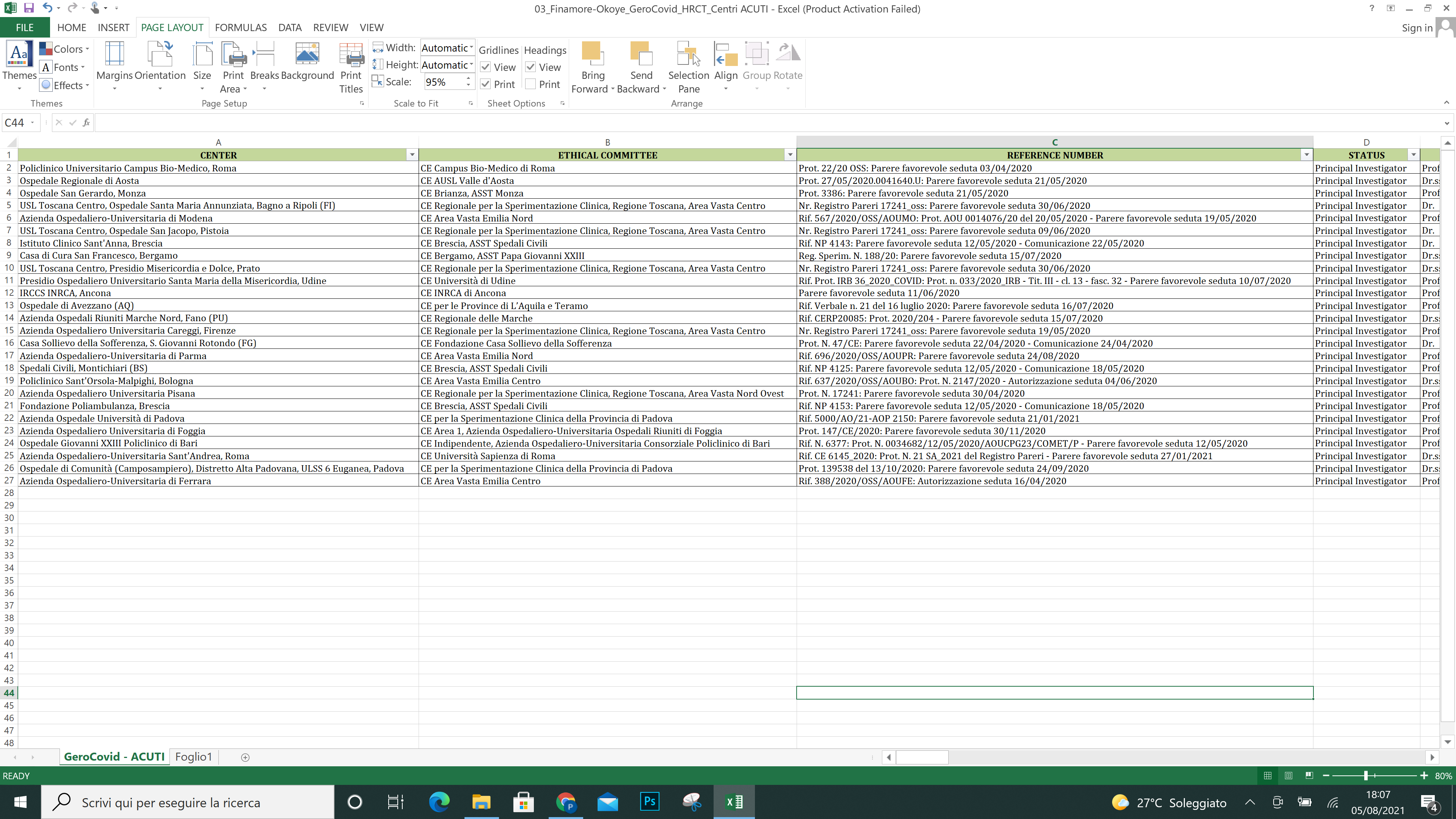

Supplement: Supplementary file 1 — Additional file 1: Summary of the IRB approvals. [file 12877_2022_2837_MOESM1_ESM.docx]
