# Supplementary material for: Computed tomography findings and prognosis in older COVID-19 patients
Source: BMC Geriatr. 2022 Mar 1;22:166. doi: 10.1186/s12877-022-02837-7 (PMC8885320; doi:10.1186/s12877-022-02837-7)
Supplement: Supplementary file 2 — Additional file 2: e-Figure 1. CONSORT flowchart diagram. e-Figure 2. Common CT-scan findings in older patients with COVID-19 pneumonia. e-Table 1. Characteristics of the GeroCovid population included (n:380) and excluded (n:820) from the analysis. e-Table2. Ground glass opacities distribution stratified by tertiles of age. e-Table 3. Distribution of CT-scan findings by clustersm. [file 12877_2022_2837_MOESM2_ESM.docx]

**Computed tomography findings and prognosis in COVID-19 older patients**

Chukwuma Okoye^1^, Panaiotis Finamore^2^, Giuseppe Bellelli^3^, Alessandra Coin^4^, Susanna Del Signore^5^, Stefano Fumagalli^6^, Pietro Gareri^7^, Alba Malara^8^, Enrico Mossello^6^, Caterina Trevisan^4^, Stefano Volpato^9^, Gianluca Zia^5^, Fabio Monzani^1^, Raffaele Antonelli Incalzi^2^

^1^ Geriatrics Unit, Department of Clinical and Experimental Medicine, University of Pisa, Pisa, Italy

^2^Geriatrics Unit, Department of Medicine, Campus Bio-Medico University and Teaching Hospital, Rome, Italy

^3^School of Medicine and Surgery, Acute Geriatric Unit, University of Milano-Bicocca, San Gerardo Hospital, Monza, Italy

^4^Geriatrics Unit and the GeroCovid Working Group, Department of Medicine (DIMED), University of Padua, Italy

^5^ Bluecompanion Ltd, London, UK

^6^Geriatric Intensive Care Unit, Department of Experimental and Clinical Medicine, University of Florence, Italy

^7^ Center for Cognitive Disorders and Dementia - Catanzaro Lido, ASP Catanzaro, Italy

^8^ANASTE Humanitas Foundation, Rome, Italy

^9^ Department of Medical Sciences, University of Ferrara, Ferrara, Italy

**Corresponding Author:**

Panaiotis Finamore, MD PhD

Unit of Geriatrics,

Campus Bio Medico University and Teaching Hospital

Via Alvaro del Portillo, 200 – Rome Italy

Ph. +39 06-225411167

e-mail: p.finamore@unicampus.it

**e-Figure 1.** CONSORT flowchart diagram.

**
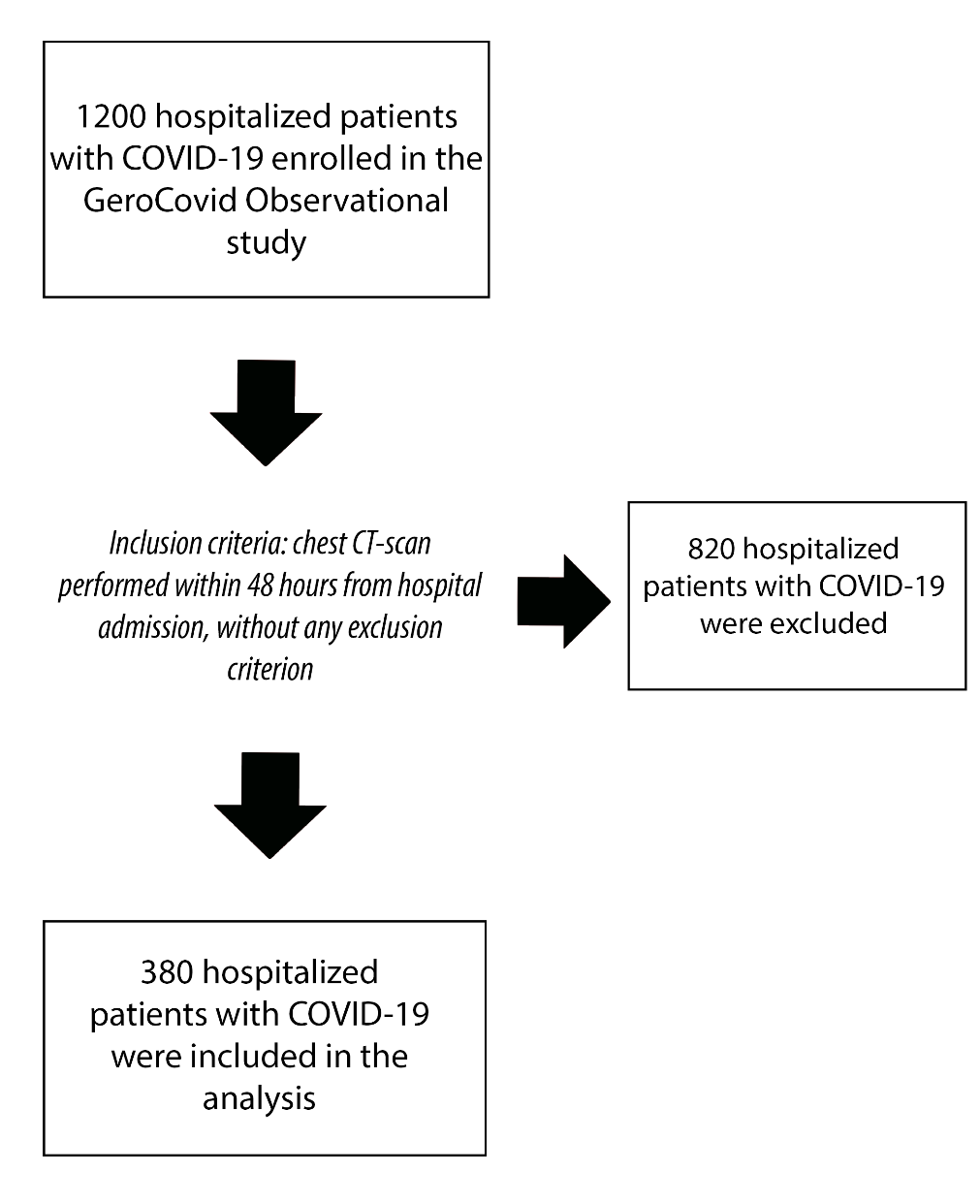
**

**e-Figure 2.** Common CT-scan findings in older patients with COVID-19 pneumonia.


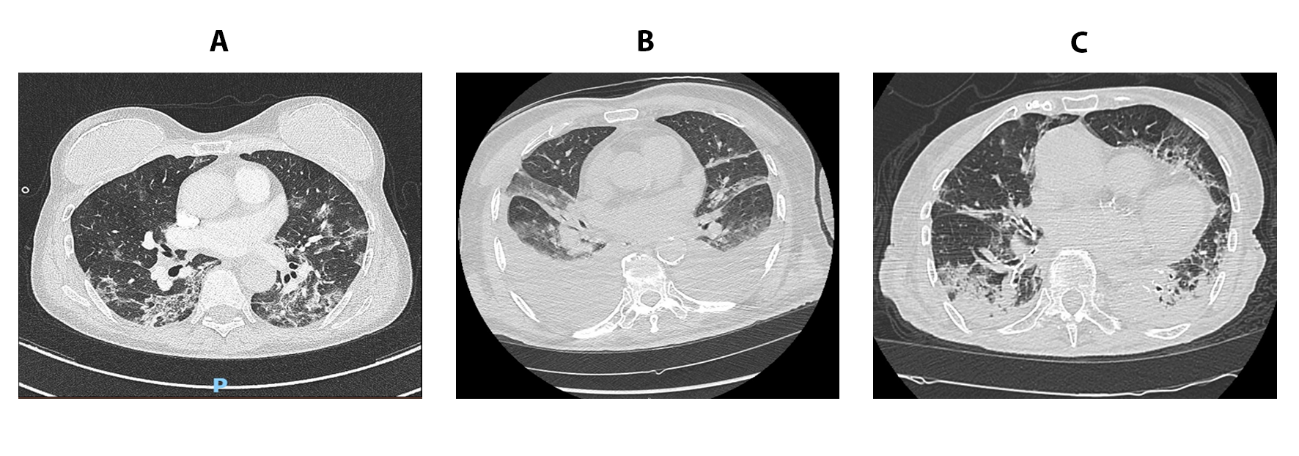


1. Bilateral posterior ground glass opacities (GGO) with perihilar patchy infiltrates of a 72-year-old woman admitted to hospital for COVID-19 pneumonia.
2. Bilateral basal and intra-scissural GGO, bilateral pleural effusion of a 81-year-old man admitted to hospital for COVID-19 pneumonia.
3. Bilateral GGO, bronchiectasis, bilateral basal consolidations and cardiomegaly in a 87-year-old man admitted to hospital for COVID-19 pneumonia.

**e-Table 1. Characteristics of the GeroCovid population included (n:380) and excluded (n:820) from the analysis.**

|  | **Population excluded (n:820)** | **Population included (n:380)** | **P-value** |
| --- | --- | --- | --- |
| **Age (years)** | 79 (7) | 78 (9) | 0.02 |
| **Gender (M)** | 402(49) | 214(56) | 0.03 |
| **Nutritional status** |  |  |  |
| Obese | 138 (17) | 60 (16) | 0.36 |
| Underweight-malnourished | 139 (17) | 48(13) | 0.89 |
| **PaO_2_/FIO_2_** | 250 (116) | 271 (110) | <0.01 |
| **Frailty** | 76(9) | 31(8) | 0.56 |
| **Pneumonia severity** |  |  |  |
| No oxygen therapy | 213 (26) | 128(34) | <0.01 |
| Oxygen by mask or nasal prongs | 336 (41) | 179(47) | <0.01 |
| NIV or HF oxygen | 246 (30) | 52(14) | <0.01 |
| Intubation and mechanical ventilation | 25 (3) | 17(5) | 0.05 |
| **Main comorbidities** |  |  |  |
| Diabetes mellitus | 183(22) | 83(22) | 0.82 |
| Chronic heart failure | 79(10) | 40(11) | 0.76 |
| Atrial fibrillation | 100(12) | 50(13) | 0.77 |
| Cognitive impairment | 182(22) | 51(13) | <0.01 |
| Hypertension | 209(26) | 83(22) | 0.15 |
| Chronic kidney disease | 80(10) | 50(13) | 0.11 |
| COPD | 69(8) | 38(10) | 0.47 |
| **Hospital stay (days)** | 3 (IQR:15) | 8 (IQR:19) | <0.01 |
| **Outcome** |  |  |  |
| Clinical improvement | 324 (43) | 198 (52) | <0.01 |
| Death | 234 (31) | 95 (25) | 0.03 |
| No major change | 56 (7) | 15 (4) | 0.02 |
| Transfer to a different hospital | 114 (15) | 72 (18) | 0.17 |

*Abbreviations:PaO2/FIO2: partial pressure arterial oxygen/fraction of inspired oxygen ratio; NIV: non-invasive ventilation; HF: high-flow; COPD: chronic obstructive pulmonary disease;*

**e-Table2. Ground glass opacities distribution stratified by tertiles of age.**

|  | **Population (n:380)** | **Aged 60-74 (n:139)** | **Aged 75-83 (n:116)** | **Aged ≥ 84 (n:125)** | **P-value** |
| --- | --- | --- | --- | --- | --- |
| **Lobe** |  |  |  |  |  |
| Upper right lobe | 137(55) | 54(54) | 36(48) | 47(64) | 0.13 |
| Middle lobe | 124(50) | 50(50) | 36(48) | 38(52) | 0.89 |
| Lower right lobe | 162(65) | 71(71) | 51(68) | 40(55) | 0.07 |
| Upper left lobe | 116(47) | 47(47) | 34(45) | 35(48) | 0.95 |
| Lower left lobe | 185(75) | 82(82) | 53(71) | 50(68) | 0.08 |
| **Distribution** |  |  |  |  |  |
| Bilateral | 239(96) | 98(98) | 69(92) | 72(99) | 0.05 |
| Subpleuric | 11(3) | 7(5) | 2(2) | 2(2) | 0.17 |
| Diffuse | 63(25) | 28(28) | 17(23) | 18(25) | 0.71 |
| **Severity score (n° of lobes)** | 2(3) | 2(4) | 2(3) | 2(3) | 0.06 |

**e-Table 3. Distribution of CT-scan findings by clusters.**

| **CT scan** | **Cluster 1 (n:103)** | **Cluster 2 (n:62)** | **Cluster 3 (n:42)** | **Cluster 4 (n:17)** | **Cluster 5 (n:156)** |
| --- | --- | --- | --- | --- | --- |
| GGO | 79(77) | 54(87) | 39(93) | 14(82) | 136(87) |
| *GGO > 2 pulmonary lobes* | 35(34) | 22(35) | 7(17) | 3(18) | 48(31) |
| Consolidation | 53(51) | 21(34) | 15(36) | 4(24) | 63(40) |
| Pulmonary nodules | 15(15) | 4(6) | 5(12) | 3(18) | 20(13) |
| Fibrosis | 7(7) | 0(0) | 2(5) | 0(0) | 9(6) |
| Bronchiectasias | 12(12) | 0(0) | 0(0) | 2(12) | 20(13) |
| Pleural effusion | 22(22) | 19(31) | 10(24) | 6(35) | 29(18) |
| Emphysema | 4(4) | 1(2) | 0(0) | 0(0) | 14(9) |
| Cardiomegaly | 7(7) | 1(2) | 3(7) | 1(6) | 7(6) |
| Pericardial effusion | 10(10) | 3(5) | 0(0) | 0(0) | 6(4) |
| Solid lesion | 10(10) | 4(6) | 1(2) | 0(0) | 4(3) |
| Pulmonary embolism | 5(5) | 0(0) | 1(2) | 0(0) | 4(3) |
| Subsegmental vascular enlargement | 1(1) | 0(0) | 0(0) | 0(0) | 0(0) |
